# ABENEARIO: A system for learning early maths with ABN

**DOI:** 10.1007/s10639-023-11692-x

**Published:** 2023-03-18

**Authors:** Ana Martín Díaz, Carlos Alario-Hoyos, Iria Estévez-Ayres, Carlos Delgado Kloos, Carmen Fernández-Panadero

**Affiliations:** Department Telematics Engineering, Universidad Carlos III de Madrid, Leganés, Madrid Spain

**Keywords:** Early math education, ABN method, Physical device, Web application

## Abstract

ABN (Abierto Basado en Números—Open Calculation Based on Numbers) is a method for teaching basic arithmetic operations in primary education that has become popular in recent years and that is based on the decomposition of numbers through manipulative materials that encourage mental calculation. Currently there is limited number of tools that can be used to support the ABN method and so this article presents the design and development of two tools that facilitate learning with this method, a physical device, ABENEARIO-P, and a virtual device (web application), ABENEARIO-V, that complements the physical device. In addition, a study of the use of these tools was carried out with 80 learners (ages 7 and 9) and 9 teachers with a focus on ABENEARIO-V. The results of this study showed a positive evaluation of the tool by both learners and teachers, an adequate time to complete the mathematical operations assigned to learners and an improvement in performance as the tool was used. As a conclusion, it is important to provide adequate tools that can support teachers and learners in the practice with the ABN method as in the case of ABENEARIO-P and ABENEARIO-V. Limitations refer mainly to the context of the study, which was conducted at a time of severe social distance restrictions during the COVID-19 pandemic on touching physical devices or being able to gather a larger number of learners in the classroom.

## Introduction

The process of teaching mathematics is highly complex (Berch & Mazzocco, 2007) and a variety of methods have been developed in recent years to try to improve its effectiveness, such as the integration of math games into math lessons, the incorporation of visual aids to represent complex concepts, or the connection of math concepts into everyday problems (Montessori et al., 2017; Stramel, 2021; Won, 2014). In the field of arithmetic, one method that has recently gained much popularity to promote the development and stimulation of basic mathematics learning at schools is the ABN method (*Abierto Basado en Números*—Open Calculation Based on Numbers) (Cerda et al., 2018). This method does not work directly with numbers. Instead, it works with digits. There are multiple ways to reach the solution of an arithmetic operations with ABN. In contrast, CBC methods (*Cerrado Basado en Cifras*—Closed Based on Ciphers) are closed and rigid (Montero, 2011), with only one correct way to do the arithmetic operations. An important characteristic of the ABN method is the emphasis on the manipulation of objects (Montero, 2001); chopsticks are typically used to represent units of the decimal system and are placed in trays where each tray represents an operand (Montero, 2018).

There is currently a limited offer of technological tools for the support of the ABN method. This article fills this gap presenting the design of ABENEARIO and its implementation as two tools from scratch, conducting a study on the use of these tools. The first tool is a physical device called ABENEARIO-P that simulates the trays used in ABN by replacing the chopsticks with LEDs (light-emitting diode). This physical device saves all the interaction that learners have with the device when solving arithmetic operations for further analysis. Due to the health situation with the COVID-19 (Coronavirus disease 2019) pandemic and the limitations for learners to use physical devices in the classroom, a second tool, in the form of a web application, called ABENEARIO-V was also developed as a complementary virtual device. ABENEARIO-V allows learners to keep track of the homework to be completed and already finished. The teacher can create classes and assign homework to learners in a personalized manner. ABENEARIO-V was evaluated through a pilot study with 80 learners from 7- to 9-year-old and 9 teachers. In this context, the following research questions are posed:

- **RQ1:** How do learners and teachers assess the usability of and experience with ABENEARIO?
- **RQ2:** Are there significant differences in learners’ performance with basic operations in ABN depending on their age using ABENEARIO?
- **RQ3:** Are there significant differences in learners’ performance with basic operations in ABN depending on the usage time of ABENEARIO?
- **RQ4:** Are there significant differences in learners’ performance with complex operations using the ABENEARIO?

## Related work

There are several educational tools in mathematics aimed at supporting learners. For example, AGILMAT is an application that focuses on the practice of calculus and algebra exercises for secondary education (Tomás et al., 2007), adapting the set of exercises to different levels and stages of learning. GeoGebra is a software tool that allows learners to practice geometry, algebra, graphics, statistics, and calculus (Hohenwarter et al., 2008), representing functions, surfaces, or volumes in a graphical way, and supporting the practice of functions, derivatives, etc. Photomath is another related application that solves equations and mathematical problems (Nguyen & Chen, 2016) using the smartphone camera to scan the problem to be solved, showing the result and the process to obtain it.

There are also tools that focus on learning basic operations for primary education. For example, EDUMAT is an application for teaching basic operations through interactive activities (Muñoz Sanabria & Vargas Ordoñez, 2019). EDUMAT was tested with 19 elementary school learners, achieving an increase in learner motivation and performance after using this application. Smartick is another tool to assist teachers in early math education with a focus on mental calculus, arithmetic, geometry, and problem solving (Marbán et al., 2018). Smartick proposes the next exercises depending on the previous performance of each learner, so every learner receives personalized exercises (Macas et al., 2020).

These tools are based on more rigid traditional methods for learning arithmetic operations (Montero, 2011) unlike the ABN method (Aragón et al., 2017). In the context of ABN, there are currently few technological tools available. One of the few related applications is “El Rincón de Luca”, a section of the “Retomates” application (Perea, 2016). “El Rincón de Luca” is a web application that offers a variety of games to work on several aspects related to ABN, such as friends of number 10, and composition and decomposition of numbers, among others. There is also a website called Penyagolosa E-Duca (E-Duca). This website organizes a series of exercises for different educational levels. These exercises have been developed using the visual programming language called Scratch (Maloney et al., 2010). Although this website offers some tools for teachers, such as organizing exercises by level, it does not collect information on learners’ progress that could be used, for example, to help learners with problems.

## The ABN method

The ABN method is used for teaching basic mathematics (Montero, 2018). This method is open, that is, it gives learners the freedom to perform arithmetic operations in the most comfortable and understandable way possible for them, with several ways of solving the same problem (Rodrigo & Fernández, 2020). The basis on which learners work with ABN is numbers. Learners use manipulative materials to acquire the concept of number. Learners work with concrete quantities, manipulate them, construct the numbers, the relations between them, compose them, decompose them, make groupings and distributions (Rodrigo & Fernández, 2020). The ABN method allows learners to apply their own strategies, in contrast to the traditional methods that treat a number as something static, determined, and closed (Montero, 2011; Montero & Cortés, 2017). The main target of the ABN method is the achievement of logic and mathematical understanding using manipulative objects (Montero & Cortés, 2017).

The operations that can be performed with ABN are addition, subtraction, multiplication, and division. There are also “new operations”: double addition, double subtraction, and addition-and-subtraction (Martínez, 2014). The most common way to work with ABN is through chopsticks. Chopsticks are grouped together to form the numbers of the operations, and each term of the operation is placed on a different tray (e.g., three single chopsticks together represent three units of the decimal system). The aim is to move chopsticks from one tray to another one until all the trays are empty except for one, which shows the result (Rodrigo & Fernández, 2020). The way in which chopsticks are moved from one tray to another depends on the operation to be carried out, but there is no single way of doing it. The learner has flexibility in deciding the order of the movements and the quantities to move. This allows learners to explore and have a better understanding of both the numbers and the possibilities to operate with them. For example, in the case of the addition the purpose is to remove chopsticks from one tray and put them in another one. In the case of the subtraction, the purpose is to remove chopsticks from one tray and remove the same number of chopsticks from the other one (Montero & Cortés, 2017). Each change from one tray to the other is considered as a different step of the operation.

The number of optimal steps for each operation can be established with the help of an expert with extensive experience on the ABN method (see examples for the basic addition and subtraction operations in Table 1). Some operations require additional steps depending on whether there is an overflow or not in the orders of magnitude. When there is an overflow in the addition, one of the summands is decomposed looking for the “friend of 10 or 100” to be able to group to a higher order (Montero & Cortés, 2017). Therefore, the addition operation needs an additional step if it has overflow. For example, the optimal number of steps for the addition “20 + 15” is 1 since the learner only must move two tens from one tray to get the result “35”, but for “53 + 9” is 2.

**Table 1:** Optimal steps for reaching the solution in several examples of addition and subtraction operations with ABN

	**Scenarios**	**Example**	**Optimal steps**
ADDITION			
Complete tens* + incomplete tens		20 + 15	1
Incomplete tens + units	No overflow	41 + 5	1
	Overflow	53 + 9	2
Incomplete tens + incomplete tens	No overflow	11 + 13	2
	Overflow in units	24 + 37	3
	Overflow in units and tens	54 + 79	3
Complete hundreds** + tens + units		200 + 20 + 6	2
Incomplete hundreds + units	No overflow	142 + 6	1
	Overflow	144 + 7	2
Incomplete hundreds + complete hundreds	No overflow	142 + 30	1
	Overflow	142 + 80	2
Incomplete hundreds + incomplete hundreds	No overflow	341 + 253	3
	Overflow in units	347 + 256	4
	Overflow in units and tens	268 + 175	4
SUBTRACTION			
Tens and units		38 – 15	2
Complete hundreds—hundreds with tens		400 – 120	2
Hundreds with tens—hundreds with tens	Hundreds and tens—hundreds and tens, where minuend tens > subtrahend tens	360 – 140	2
	Hundreds and tens—hundreds and tens, where minuend tens < subtrahend tens	360 – 180	3
Complete hundreds—incomplete hundreds		500 – 156	3
Incomplete hundreds—incomplete hundreds		376 – 186	3

* Complete tens: multiples of 10 up to 90.

** Complete hundreds: multiples of 100 up to 900.

Operations with three terms, i.e., complex operations, can be divided into two basic operations to establish the optimal number of steps. For example, addition-and-subtraction is a union of an addition and a subtraction. Two operations and three trays are used, so this complex operation is more difficult and offers many variants. Therefore, when learners are introduced to complex operations they need to be in an advanced state of proficiency with respect to basic operations. The number of optimal steps for each complex operations can also be established with the help of an expert teacher, considering that learners tend to use at least five steps in solving this type of operations.

### Example of operation in ABN: Addition

**Fig. 1:**
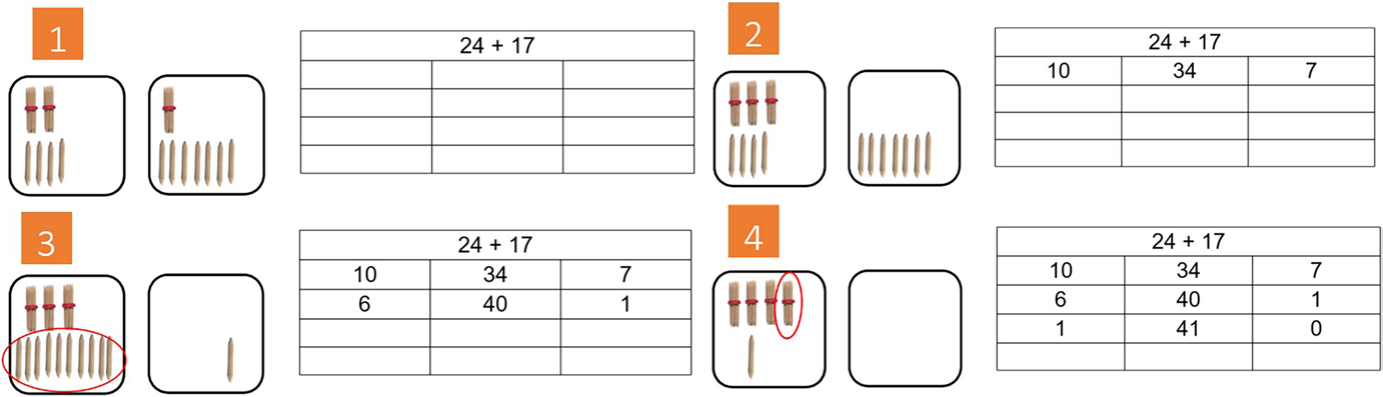
Steps to calculate the addition of “24 + 17” using chopsticks, trays, and a grid with the ABN method

The addition operation consists of removing a certain amount from one summand of the operation and adding that same amount to the other one. Learners initially use chopsticks to perform these actions. Therefore, addition involves taking chopsticks from one tray and putting them in the other tray. The solution of the operation is obtained when one of the trays is empty. Figure 1 shows a step-by-step example of how a learner would perform an addition using the chopsticks, the trays, and a grid. First, the learner represents the operands numerically on the grid or manipulatively in the trays using the corresponding chopsticks. The example of Fig. 1 (step 1) shows that 2 tens and 4 units were placed in the left tray, and 1 ten and 7 units were placed in the right one, 24 + 17. Then, the learner passes the ten from the right tray to the left one. The amount that the learner decides to pass from one side to the other one must be placed in the left column of the grid, and the terms of the operation are updated in the middle and right column, in this case, 34 + 7 (Fig. 1 (step 2)). Next, the learner finds that there are 4 “single” units in the left tray and 7 in the right one. The next step could be done in several ways, for example, trying to group 10 units to form 1 ten. In this case, the learner moves 6 units from the right tray to the left one. Hence, there are 10 units in the left tray that the learner can group to 1 ten. In addition, the terms of the operation are updated in the grid, 40 + 1 (Fig. 1 (step 3)). Finally, the learner moves the last unit from the right tray to the left one. Now, one tray is empty and therefore, the solution was found. This change is also indicated in the grid (Fig. 1 (step 4)).

## Methodology

This section presents the methodology used, starting with the design of ABENEARIO as a tool to support the learning and practice of the ABN method and its development as a physical device (ABENEARIO-P) and the equivalent virtual device (ABENEARIO-V). The data collection and analysis process used to evaluate ABENEARIO are presented below, detailing the variables and techniques used. Finally, the experiment carried out to evaluate ABENEARIO and answer the research questions are presented.

### ABENEARIO-P: Physical ABN device

**Fig. 2:**
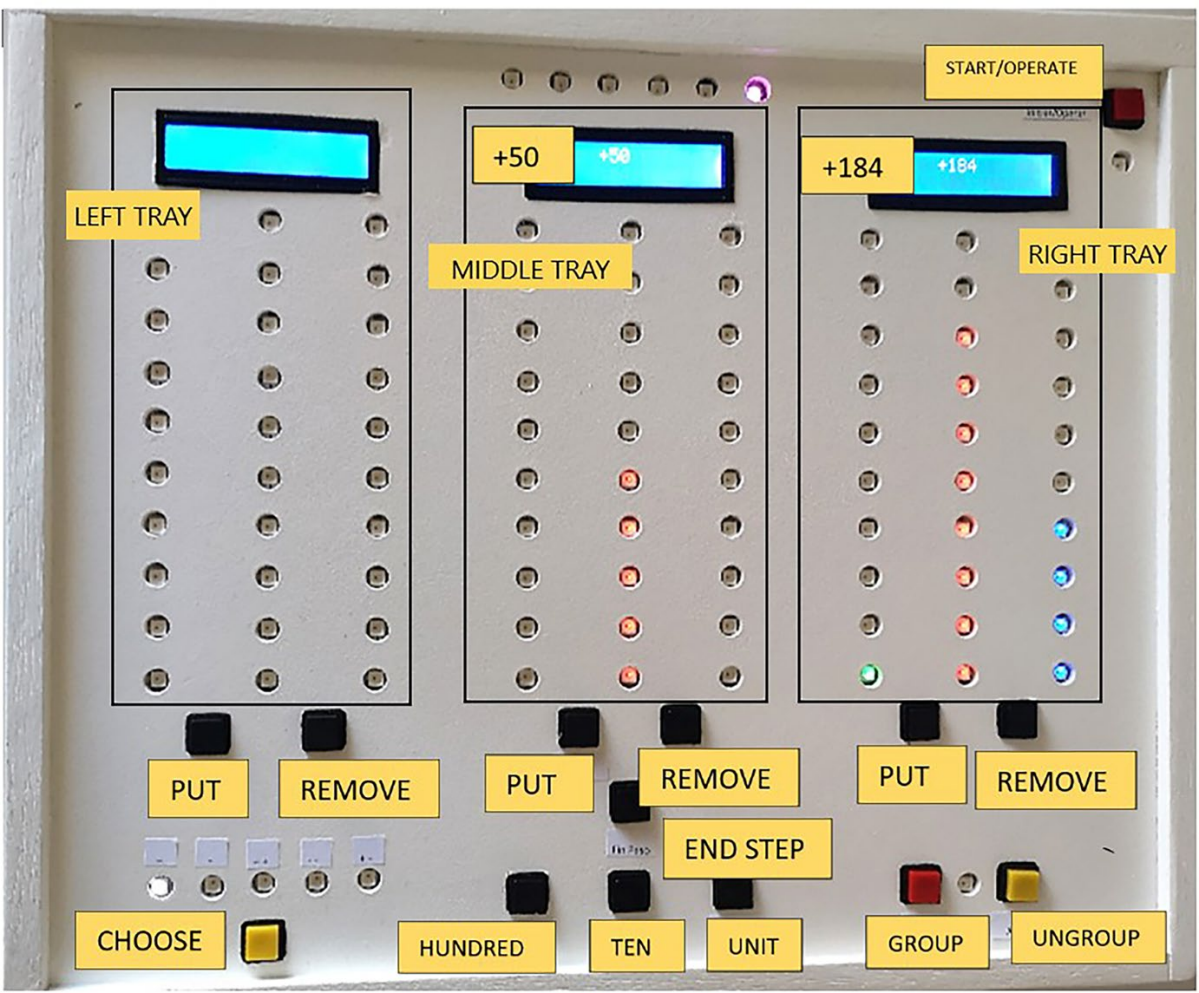
Physical ABN device: Example of an intermediate step of an addition operation with the middle tray containing the value 50 and the right tray containing the value 184

A physical device was implemented to support learners in the process of learning basic arithmetic operations with the ABN method and with the objective of being able to collect information on the iterations that occur between the learner and the physical device for a further analysis. The physical device was designed with the aim of preserving the main characteristics on which the ABN method is based (Fig. 2). The physical device has three trays to represent operations up to three terms (Fig. 2-Left/Middle/Right Tray). The traditional grid used in ABN is simulated in this device by three sets of LEDs and each LED represents one chopstick, i.e., one unit, one ten, or one hundred of the decimal system. Thus, a set of LEDs consists of three strips with ten LEDs each. One set of ten LEDs is illuminated in blue to represent the units, a second set of ten LEDs in red to represent the tens, and a third set of ten LEDs in green to represent the hundreds. Each tray has its own LCD (liquid–crystal display) screen, which displays the decimal number represented by the illuminated LEDs on the trays corresponding to that moment, and the symbol for each operation (+ addition,—subtraction).

Therefore, ABENEARIO-P makes it possible to represent numbers physically, just as learners do with chopsticks, and to perform two- and three-digit operations (addition, subtraction, double addition, double subtraction, and addition-and-subtraction). Multiplication and division are not performed in trays, so they were not included in the physical device. The operation to be done is selected by pressing a button that illuminates the LED next to the name of each of the five possible operations (Fig. 2-Choose). The button moves the illuminated LED one position to the right every time it is pressed. The selected operation is the one determined by the illuminated LED.

Once the operation to be performed is selected, the “start/operate” button can be pressed and a LED will turn on (Fig. 2-Start/Operate). This means that the learner can set the operands. Then, the learner presses the “start/operate” button again and the LED will turn off, meaning that the operation is ready to be performed. There are two buttons under each tray: one to “remove” and another one to “put” (Fig. 2-Put/Remove). When learners want to move quantities from one tray to another, they must press the corresponding button. The specific quantity learners want to move is determined by the number of times they press the “unit”, “ten” and “hundred” buttons in the center of the device (Fig. 2-Unit, Ten, Hundred). Another button indicates the end of the step (Fig. 2-End Step). This button also checks that the changes between trays are consistent. The LEDs on the trays remain on amber color until the “end step” button is pressed and the consistency of the changes is validated.

The concepts of “group” and “ungroup” are important in the ABN method, so two buttons are included to perform these actions (Fig. 2-Group/Ungroup). When one of the LED strips is completely illuminated, an order of magnitude is completed. Consequently, a LED located between these buttons turns on alerting the learner. If the group button is pressed, the ten LEDs will turn off and a LED of the higher order of magnitude will light up. The learner cannot have a complete order of magnitude and not group the elements in this order of magnitude into the higher order, that is why the LED lights up to alert the learner. However, the user is not alerted in the case of an “ungroup” action. This is voluntary and the “ungroup” button must be pressed when the learner needs it.

Furthermore, six LEDs are placed on the top of the device to indicate the number of steps done while the learner performs the operation. If the learner needs more steps than the ones considered as optimal, the LED lights up in a different color. Thus, the teacher can identify if there is a learner who needs help. Finally, the device saves learners’ interactions while performing the arithmetic operation so that this data can eventually be used in the future to provide more information to the teacher and the learner about the process followed.

The hardware implementation of the device was done with the Arduino Mega development board, RGB (Red, Green, Blue) LED strips, and buttons. The software development was used the corresponding platform for Arduino, Arduino IDE (Integrated Development Environment). The data collected from learners’ interaction with the device is stored in JSON (JavaScript Object Notation) format.

### Example of operation in ABN with ABENEARIO-P: Addition

The addition operation detailed in section “Example of Operation in ABN: Addition” would be done as follows on the physical device. First, the learner chooses the operation to be performed by pressing the “Choose” button (Fig. 2-Choose). Once the illuminated LED is on the addition option, the learner presses the “Start” button to set the operands (Fig. 2-Start/Operate). To do this, the learner presses the “Put” button of the tray where each term of the operation (24 + 17) is to be placed, followed by the “Unit” and “Ten” buttons, which should be pressed as many times as units and tens each term has. The learner presses the “Operate” button when the trays have the corresponding LEDs illuminated (Fig. 2 -Start/Operate). Next, the learner presses the “Remove” button on the right tray and removes a ten by pressing the “Ten” button once. The learner has to put that ten on the left tray, so the learner presses the “Put” button on the left tray and then presses the “Ten” button once. The learner presses the “End Step” button to check that the changes were made correctly and now, the terms in each tray are 34 + 7. Then, the learner removes 6 units from the right tray and puts them on the left one. For this, the learner presses the “Remove” button on the right tray and presses the “Unit” button 6 times. Then, the learner presses the “Put” button on the left tray and presses the “Unit” button 6 times again. The learner presses the “End Step” button and the terms of the operation are 40 + 1. Now, there are 10 full units in the left tray. Therefore, the LED next to the “Group” button lights up to alert the learner to perform this action. The learner presses the “Group” button so that the 10 units go out and 1 LED for the tens lights up. Finally, the learner removes the last unit from the right tray and places it on the left one. One tray has all its LEDs off, so the solution has been found.

### ABENEARIO-V: Virtual ABN device

**Fig. 3:**
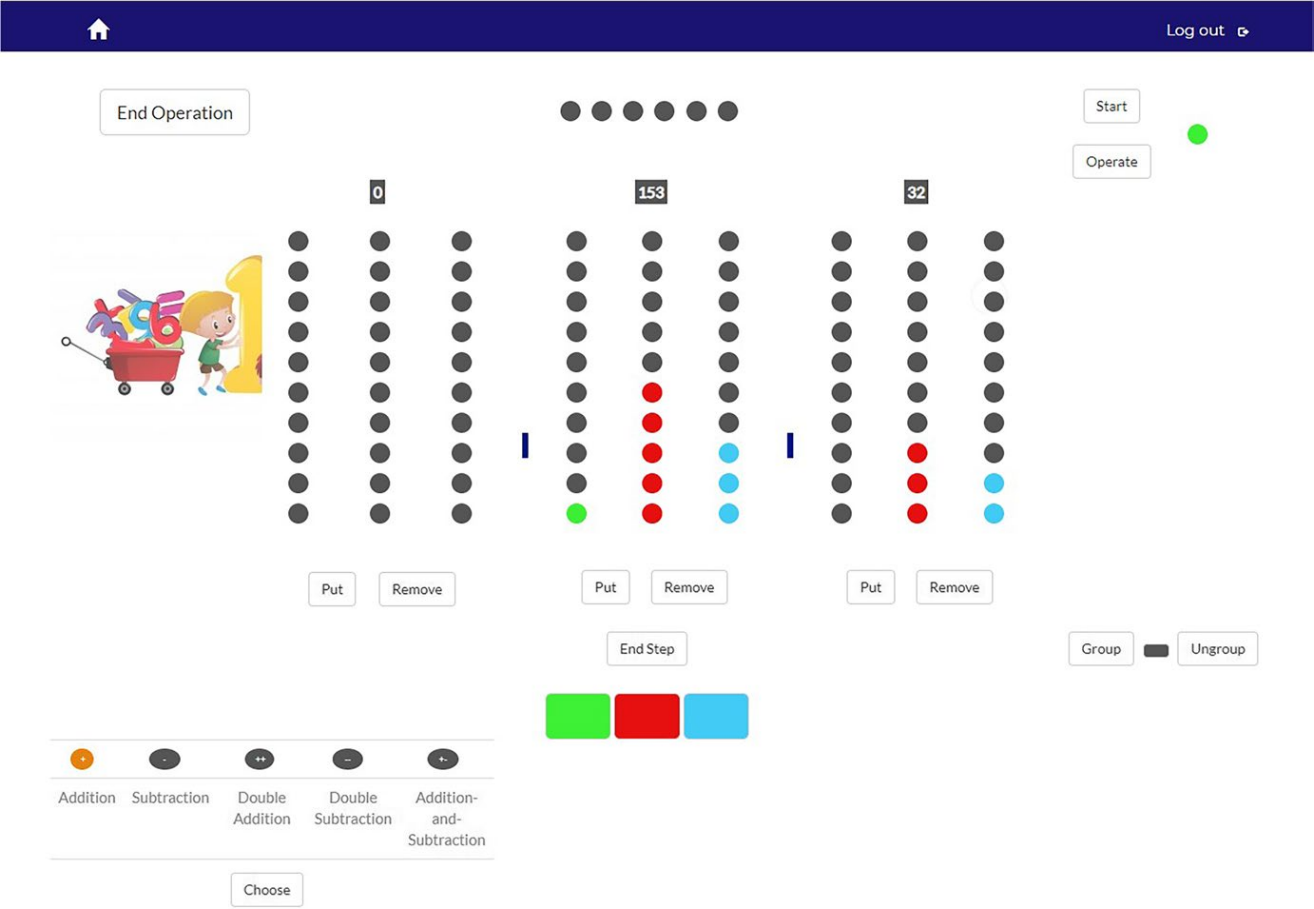
Virtual ABN device as a web application that replicates the physical ABN device

**Fig. 4:**
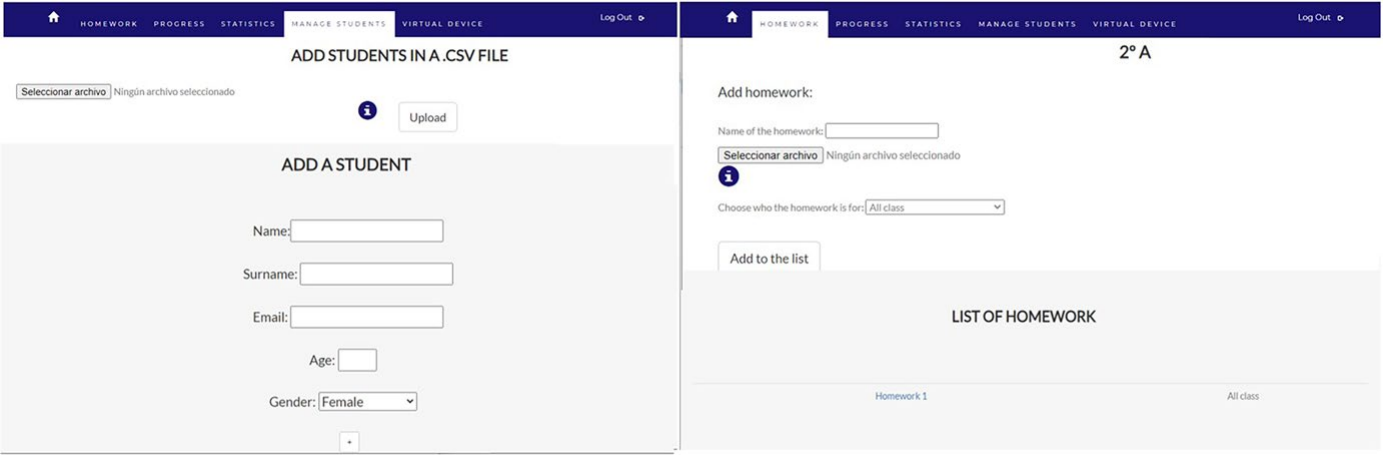
Interfaces for managing learners (left) and homework (right) in the ABN web application (teacher role)

A virtual device (web application) called ABENEARIO-V was implemented with the aim to simulate the behavior of the physical device (Fig. 3), supporting the same type of operations, but expanding some of the functionalities of ABENEARIO-P, considering two roles:

- **Teacher Role.** When teachers log in, their main page is the management of their classes. They can create a new class, see the list of classes already created and select the one they want to access. Once teachers select a specific class, they see the page to manage their learners. The teacher is the only one who can register learners in the class. Teachers can add individual learners or a list of them (Fig. 4-left). Teachers can view the list of learners and their general information. Teachers can also define tasks with a list of arithmetic operations and assign them to the whole class or to a specific learner (Fig. 4-right).
- **Learner Role.** Learners have two pages as users. One of them contains the replica of the physical ABN device (Fig. 3). The procedure of performing an operation is the same as in the physical device. Additionally, there is a section for learners to check the homework that the teacher assigned to them.

ABENEARIO-V (web application) is based on the MVC (Model-View-Controller) pattern (Mak, 2008). The Model component manages the storage, retrieval, and manipulation of application data in a database using MySQL (My Structured Query Language). The View component collects user input and produces HTML (HyperText Markup Language) output (using the Thymeleaf template language (Reddy, 2017)), which is sent to the user. The style is deployed using CSS (Cascading Style Sheets) and JavaScript code. The Controller component coordinates the application: it receives user input from the view, acts on the model to process it and communicates to the view the data to be displayed to the user. Finally, it should be noted that the logic to replicate ABENEARIO-P was implemented using JavaScript in ABENEARIO-V.

### Data collection and analysis process

The evaluation of ABENEARIO is aimed to answer the research questions and requires first to define the sources of information from which data is collected, the variables to be analyzed and the data analysis techniques to be applied to these variables.

#### Data collection

Several information sources allow the collection of data when experimenting with the use of ABENEARIO (Table 2): information collected directly by ABENEARIO [ABNLogs]; surveys completed by learners [LearnerSurvey] (Appendix A) and teachers [TeacherSurvey] (Appendix B with the SUS—System Usability Scale—questionnaire (Brooke, 1996) and some additional questions); and observations by teachers [TeacherNotes] and researchers [ResearcherNotes].

**Table 2:** Data collection techniques

Data Source	Type of Data	Label
ABENEARIO logs	Data collected by the tool	[ABNLogs]
Learners Survey	Questionnaire with demographic data open and closed questions	[LearnersSurvey]
Teachers Survey	SUS (System Usability Scale) questionnaire (Brooke, 1996)	[TeachersSurvey]
Field observation from teachers’ practice	Observations collected by the teachers	[TeacherNotes]
Field Observation from researchers’ practice	Observations collected by the researchers	[ResearcherNotes]

#### Variables

The variables under study are:

- **Age.** This is an independent variable representing the age of each learner.
- **Average number of steps.** This is a dependent variable representing the average number of steps a learner needs to complete a mathematical operation. A new step is defined as each time the learner presses the button *“End Step”*.
- **Average time.** This is a dependent variable that represents the average time that a learner takes to finish an operation. Each event in ABENEARIO has its associated timestamp. Therefore, the time to complete an operation is calculated by subtracting the timestamp of the first event from the timestamp of the last event.

#### Data analysis techniques

The following techniques are used for the data analysis:

- **T-test.** The t-test for independent samples is used to determine whether there is a significant difference between the means of two groups. The samples must fit a normal distribution. If the number of samples is greater than 30, by the Central Limit Theorem it can be stated that the sampling distribution of the mean is normal (Rosenblatt, 1956). Also, it is necessary to check the homogeneity of variances in the two groups of samples (Levene’s test).
- **Welch’s t-test.** This test is used to determine whether there is a significant difference between the means of two groups when the variance homogeneity assumption (Levene’s test) is not fulfilled, as an alternative to the t-test.
- **Mann–Whitney test.** This non-parametric test is used when the data does not meet the requirements of the t-test. The Mann–Whitney test is useful in the case of two independent samples where the purpose is to check if there is a difference in the magnitude of the variable under study. This test is used to analyze the samples when normality cannot be assumed by the central limit theorem, and the Shapiro-Wilks test is not accepted.
- **Kruskal–Wallis test.** This non-parametric test is used to test whether a data set is from the same population when normality cannot be assumed for that data set.

The null hypothesis in the cases of t-test, Welch’s t-test and Mann–Whitney test is that the mean [time / number of steps] for performing an [addition / subtraction] in [age group 1 / age group 2] learners is the same, while in the case of the Kruskal–Wallis test is that the mean [time / number of steps] for the double addition, double subtraction, and addition-subtraction for the learners of the same age group is the same.

### Evaluation of ABENEARIO

A two-day workshop was organized in a primary school in Spain. Learners from Primary Education belonging to two age groups of 7 (second year of Primary Education) and 9 (fourth year of Primary Education) whose teachers agreed to participate in the study had a first session to practice the ABN method with ABENEARIO. There were 80 learners in total who were already familiar with the ABN method, 43 were 7 years old (20 female and 23 male) and 37 were 9 years old (22 female and 15 male). Each session was conducted by a teacher with extensive expertise on ABN. The responsible teacher for each class was present as well. Learners of both ages completed the same operations so that a comparative analysis between ages could be carried out later with the data generated. The operations that learners performed during each session are shown in Table 3. In addition to the two-day workshop, an additional session was done with the 9-year-old learners from the same school two weeks later (37 learners in total, 22 female and 15 male). The session was guided by one of their teachers but in this case, as learners were already familiar with the tool, the purpose was to allow them to practice all the types of operations that ABENARIO could support.

**Table 3:** Operations learners performed in each session

Type of operation	Session 1	Session 2	Order in which the operations were carried out
Addition	327 + 284	347 + 475	1
	163 + 148	785 + 47	2
	-	647 + 185	3
Subtraction	316 – 98	617 – 249	4
Double Addition	-	164 + 236 + 35	5
Double Subtraction	-	75 – 13 – 48	6
Addition-and-Subtraction	-	164 + 236 – 325	7
		86 + 37 – 48	8

## Results

The health situation with COVID-19 pandemic and the restrictions on touching physical devices prevented the study from being conducted with ABENEARIO-P, for which only a demonstration to the class could be carried out with the participation of two learners. Instead, ABENEARIO-V was used for collecting data aimed at answering the research questions, as both ABENEARIO-P and ABENEARIO-V had an equivalent functionality. ABENEARIO-V could be used by 80 learners and 9 teachers. This section is divided into four parts to address the proposed research questions. RQ1 shows the results of the validation of ABENEARIO-V from the point of view of usability. RQ2, RQ3 and RQ4 support the results obtained in RQ1 by analyzing real data of learners using ABENEARIO-V.

### RQ1: How do learners and teachers assess the usability of and experience with ABENEARIO?

A survey was filled in by learners (80) and another one by teachers (9). 62 learners stated that they felt very excited using the web application on their tablets [LearnersSurvey] (Table 4). In addition, learners were asked if they would have liked to use the physical device (Table 5, 2) with 65 of them agreeing on this. 73 learners also agreed that they would like to use these tools in the classroom (Table 5, 3). Finally, they were asked to rank the tools (Table 6). The highest score was for the web application, with 73 choosing it as their favorite tool, then the physical device, and finally the chopsticks. Learners also emphasized that they really enjoyed using the virtual device. They also indicated that they found it easy and fun.

**Table 4:** Answers regarding learners’ perception

	Q1*	
Really good	62	77.5%
Good	14	17.5%
Neither good nor bad	4	5%

* How do you feel using the ABN web application.

**Table 5:** Answers regarding learners’ interests

	Q2*		Q3**	
Agree	65	81.25%	73	91.25%
Neither agree nor disagree	13	16.25%	6	7.5%
Disagree	2	2.5%	1	1.25%

* Would have you liked to use the ABN device?

** Would you like to use some of these tools in your classroom?

**Table 6:** Learners’ preference (1-least preferred, 3-most preferred)

	1	2	3
Chopsticks	73 (91.25%)	5 (6.25%)	2 (2.5%)
Device (ABEANEARIO-P)	7 (8.75%)	71 (88.75%)	2 (2.5%)
Web application (ABENEARIO-V)	0 (0%)	4 (5%)	76 (95%)

* ABENEARIO-V and chopsticks were used by all students who participated in the study. In the case of ABENEARIO-P only a demonstration was done due to the restrictions for touching physical devices derived from COVID-19 pandemic.

The SUS questionnaire was used to measure the usability of the web application in the case of teachers. Questions are in a range of 1 to 5 in the SUS questionnaire but a conversion system is used so that the score is represented in the range of 0 (lowest) to 100 (highest). The results showed an average score of 94.06. All the teachers stated that this web application is useful for their learners and that they would use it in their classes. Some of the comments they made about ABENEARIO were that they find it a motivating and effective resource for learners, as it captures their attention and they learn by playing. Furthermore, they highlighted its visual aspect and the good reflection of the manipulation characteristic of the ABN method. They also suggested that the web application could be extended to more digit operations.

### RQ2: Are there significant differences in learners’ performance with basic operations in ABN depending on their age using ABENEARIO?

**Fig. 5:**
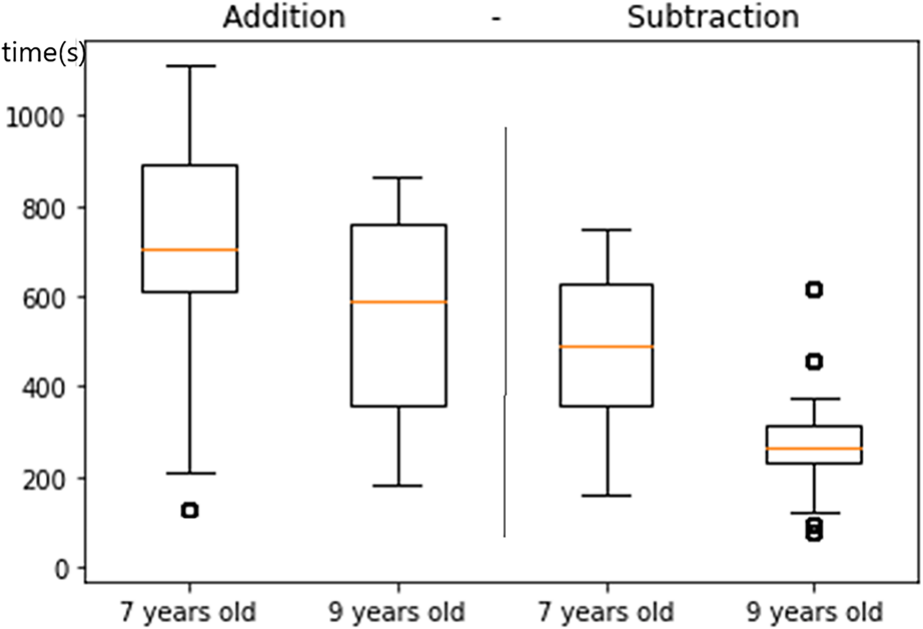
Comparison of the average time (in seconds) for learners of 7 and 9 years (addition and subtraction)

Low-level data [ABNLogs] and observations [TeacherNotes][ResearcherNotes] were used. The operations analyzed in this question were the two additions (327 + 284, 163 + 148) and the subtraction (316–98) performed in session 1. Average time and average number of steps were analyzed for learners of ages 7 and 9 years. For the average time that learners of different ages took to complete the same additions the t-test for independent samples was used. The result obtained was *p*_*value* = 0*.*2223*, t*_*statistic* = 1*.*2289 [ABNLogs]. Therefore, there was no statistically significant evidence to reject that the average time taken by 7- and 9-year-old learners to complete an addition is the same. In the case of the subtraction the result obtained was *p*_*value* = 2*.*7575*e^−10^*, t*_*statistic* = 7*.*2454. In the case of subtraction there was significant evidence to reject the hypothesis. Learners aged 7 and 9 did not spend the same average time on performing a subtraction. Figure 5 shows how the average time taken to perform an addition and a subtraction was distributed depending on the age.

**Fig. 6:**
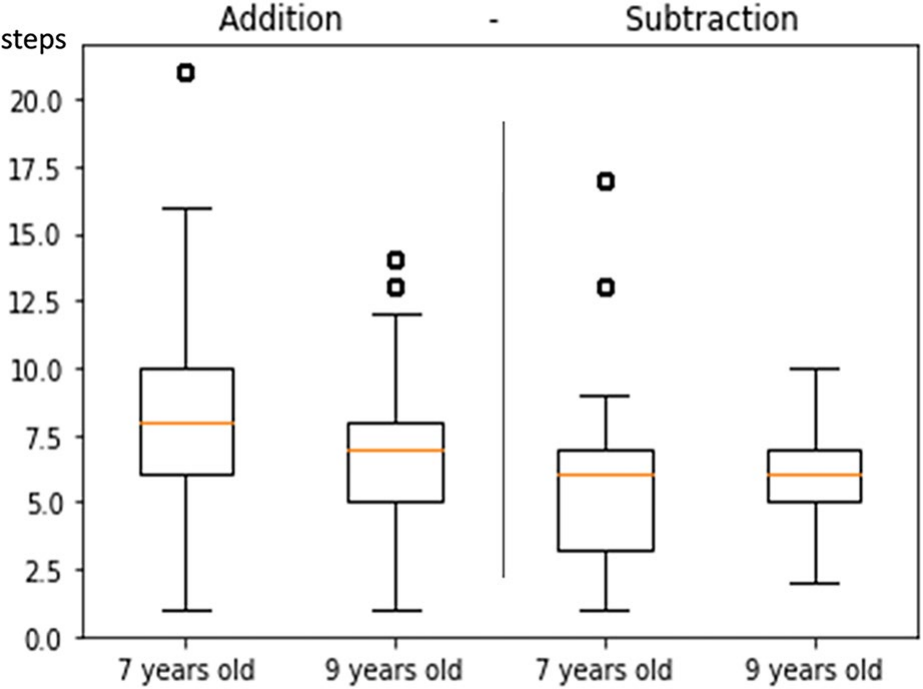
Comparison of the average number of steps for learners of 7 and 9 years (addition and subtraction)

The results in the case of the number of steps used [ABNLogs] to perform the same operations were *p*_*value* = 0*.*3817*, t*_*statistic* = 0*.*8791 for the case of the addition and *p*_*value* of 0*.*5088 and *t*_*statistic* = -0*.*6637 for the case of the subtraction. This means that there was no statistically significant evidence to reject that learners aged 7 and 9 took the same average number of steps to perform an addition nor a subtraction. The distribution of the average number of steps used to perform the operations can be seen in Fig. 6.

Finally, a comparison was made of the average time taken and steps used to perform the additions and subtraction between learners of the same age [ABNLogs]. 7-year-olds did not take the same average number of steps to complete an addition as they did to complete a subtraction (*p*_*value* = 0*.*0116*, t*_*statistic* = 2*.*5673). The average time was neither the same (*p*_*value* = 0*.*0017*, t*_*statistic* = 3*.*2279). In the case of 9-year-old learners the average time was not the same (*p*_*value* = 2*.*8355*e^−7^*, t*_*statistic* = 6*.*1742), but there was no statistically significant difference to reject that the average number of steps was the same (*p*_*value* = 0*.*3241*, t*_*statistic* = 0*.*9961).

It is noteworthy that the average time to perform an addition was longer than the average time to perform a subtraction for both ages. Based on [ResearcherNotes], this could be related to the fact that learners started the first session doing additions, and this was the first contact they had with the virtual device. According to the optimal steps established for addition and subtraction, the additions learners performed in the first session could be solved with 4 steps, and the subtraction with 3 steps. Figure 6 shows that the average number of steps used in both cases was higher than the optimal number of steps.

### RQ3: Are there significant differences in learners’ performance with basic operations in ABN depending on the usage time of ABENEARIO?

**Fig. 7:**
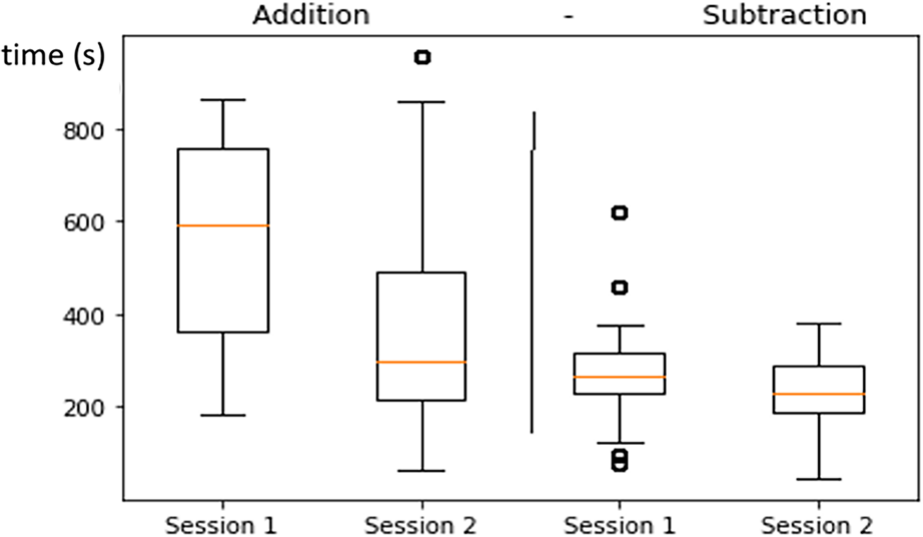
Comparison of the average time (in seconds) for 9-year-old learners between sessions

**Fig. 8:**
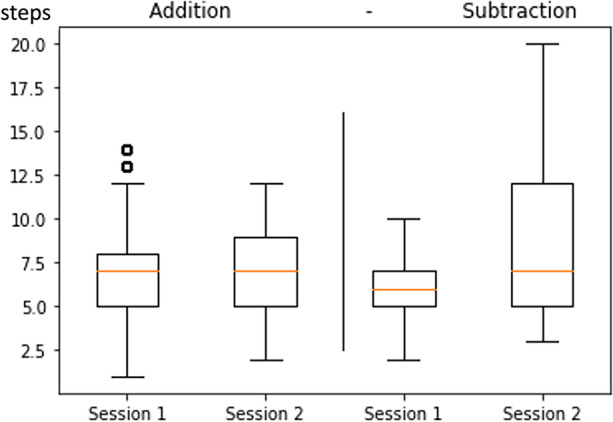
Comparison of the average number of steps for 9-year-old learners between sessions

The average time and number of steps 9-year-old learners took to complete an addition and a subtraction in the first and second sessions were compared (Figs. 7 and 8). The data used for this analysis were low-level data [ABNLogs] and observations [TeacherNotes], [ResearcherNotes]. The Mann–Whitney test was used for this comparison. The operations done were the two additions (327 + 284, 163 + 148) and the subtraction (316–98) from session 1, and three additions (347 + 475, 785 + 47, 647 + 185) and a subtraction (617–249) in session 2.

The average time required to complete an addition in the sessions was not the same [ABNLogs] (*p*_*value* = 0*.*0005, *u*_*statistic* = 216*.*5). Figure 7 shows that the time taken for the addition in the first session was much higher. This might be explained by the fact that the addition was the first operation learners did with the virtual device. However, the null hypothesis could not be rejected in the case of the average number of steps (*p*_*value* = 0*.*3485*, u*_*statistic* = 408). The same analysis was done for the case of the subtraction. The average time taken to solve a subtraction in each session was not the same (*p*_*value* = 0*.*045*, u*_*statistic* = 238). Also, the average number of steps learners needed to complete subtractions was not the same (*p*_*value* = 0*.*0075*, u*_*statistic* = 198).

These results confirm that ABENEARIO was an easy-to-use tool, as learners, after only one session and two weeks later remembered how to use it and even improved their performance. However, the amount of time the device was used did not influence the number of steps used to solve the operations.

### RQ4: Are there significant differences in learners’ performance with complex operations using the ABENEARIO?

The average time and number of steps were compared for three complex operations: double addition, double subtraction, and addition-and-subtraction. The Kruskal–Wallis test was used for this comparison. The operations analyzed in this question were performed in the second session: 164 + 236 + 35, 75–13-48, 164 + 236–325, and 86 + 37–48.

**Fig. 9:**
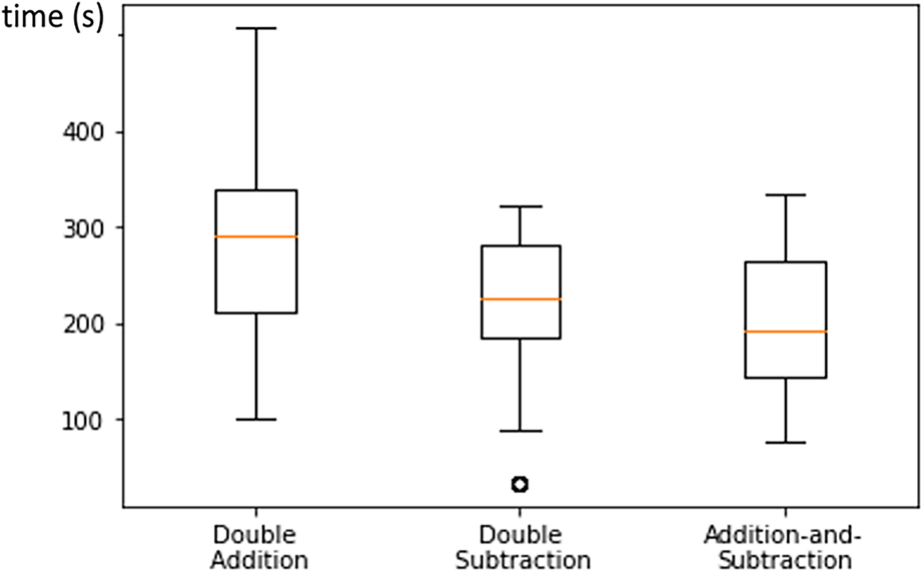
Comparison of the average time (in seconds) for 9-year-old learners to perform complex operations

**Fig. 10:**
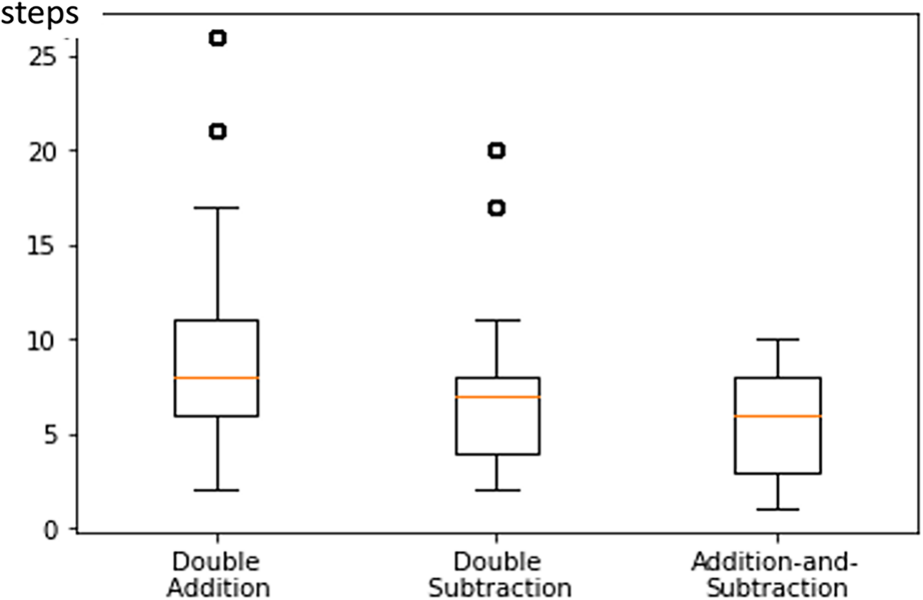
Comparison of the average number of steps for 9-year-old learners to perform complex operations

There was no significant evidence to claim that the average time taken to solve the three types of operation was the same (*p*_*value* = 0.0098, *h*_*statistic* = 9*.*2482) [ABNLogs]. Figure 9 shows that the average time in the case of double addition was higher than for the rest of the operations. The average number of steps that learners needed to solve each operation was the same (*p*_*value* = 0.0297, *h*_*statistic* = 7*.*0358). Double addition took higher average number of steps than double subtraction and addition-and-subtraction (Fig. 10).

Not all learners were able to complete all operations; only the most skilled learners completed double subtraction and addition-and-subtraction [TeacherNotes], [ResearcherNotes]. The optimal number of steps that was established to solve the double addition and the double subtraction was 3. This number was lower than that from the results obtained, although it was observed that the average number of steps that learners used in the double subtraction was lower than in the case of the double addition (Fig. 10). The optimal number of steps established for double subtraction was 5 and the results obtained were closer to this value.

## Discussion

In relation to the first research question (RQ1), it is possible to state that the use of ABENEARIO (in particular of ABENEARIO-V), provided a positive experience for learners according to their own perception. This is supported by the SUS, with an average score of 94.06, which can be interpreted as excellent (Bangor et al., 2008). It is important to highlight that this instrument has been extensively used in the literature to evaluate learners’ perceived usability of educational technology (Vlachogianni & Tselios, 2022). Learners also expressed their interest in using ABENEARIO in the classroom to practice the ABN method, revealing their preference for the use of ABENEARIO (in its virtual and physical versions) versus the use of the traditional chopsticks. In any case, it would be necessary to extend the study that was carried out to avoid the novelty effect in the use of technology (Jeno et al., 2019). Teachers also assessed positively ABENEARIO in terms of usability using the SUS as instrument.

In relation to the second research question (RQ2), it is possible to state that there were significant differences between learners aged 7 and learners aged 9 when performing the subtraction operation with ABENEARIO in terms of the time used (but not in the case of the addition operation). There were also differences in the average time required to perform the subtraction operation versus the addition operation (less time in the latter) for learners of the same age. The number of steps for both the addition and subtraction operations was higher than the optimal number of steps defined by the expert teacher; the practice with ABENEARIO and the information gathered by the tool could help to try to approximate the number of steps used by learners to the optimal number of steps.

In relation to the third research question (RQ3), it is possible to state that there are differences in the time needed to complete addition and subtraction operations as the time of use with ABENEARIO increases (less time is needed as the time of use increases). Despite the time elapsed between sessions, this time reference continued to improve. Moreover, the fact that considerable time elapsed between times of ABENEARIO use was not detrimental to the result obtained.

In relation to the fourth research question (RQ4), it is possible to state that the double addition takes more time than the other complex operations (addition-subtraction, double subtraction) although it should always be borne in mind that only the most skilled learners could perform these more complex operations (Montero, 2018), which introduces a certain bias in the results obtained for this last research question.

## Conclusion and future work

This work presented the development of a physical device (ABENEARIO-P) and the equivalent web application (ABENEARIO-V) for learning early math using the ABN method. A two-day workshop was organized with 80 learners and 9 teachers who used the application. Learners’ evaluation of ABENEARIO-V was very positive. The majority preferred it over the traditional chopsticks. Besides, learners would like to use ABENEARIO in their classes. Teachers very positively assessed the usability of ABENEARIO-V according to the SUS questionnaire. Learners’ performance with the ABENEARIO-V also supports this. The time learners needed to complete the operations was adequate. Learners improved their performance two weeks after the workshop. This means they easily remembered how to use the tool.

Limitations of this work include the working context. The evaluation was done in a period of severe restrictions due to the COVID-19 pandemic, which limited the number of learners using ABENARIO-P and the usage time of ABENEARIO-V as learners could only use the web application in the classroom under teachers’ supervision. The functionality of ABENEARIO-V could be extended as suggested by the teachers, for example, to work with numbers with more than three digits, and the possibility of practicing more operations. Future work considers the development of an intelligent tutor for ABENEARIO to suggest exercises and the optimal sequence of steps to solve an operation. In addition, while currently ABENEARIO captures learners’ interactions when solving each arithmetic operation (in both its physical and virtual versions), it is not yet provided a dashboard to show teachers their learners’ progress and so this is a functionality to be developed in the near future. In the same way learners should have an option that allows them to get personalized feedback from their teachers.

## Data Availability

Most of the data generated or analyzed during this study are included in this published article (and its supplementary information files). The datasets generated during and/or analyzed during the current study are available from the corresponding author on reasonable request.

## Appendix A. Learners questionnaire

1. Indicate your course.
2. How do you feel using the ABN web application on your tablet? [Really good—Good—Neither good nor bad—Bad—Really bad]
3. Would have you liked to use the ABN physical device? [Agree—Neither agree nor disagree—Disagree]
4. Would you like to use any of these tools in your classroom? [Agree—Neither agree nor disagree—Disagree]
5. Which would you prefer to use? Rank the tools you would like to use, being 1 the one you like the least and 3 the one you like the most. [Chopsticks—Device—Web Application]
6. Please give details of what you liked best. [Free text]
7. Please detail what you liked the least. [Free text]

## Appendix B. Teachers questionnaire SUS questionnaire

1. I think that I would like to use this system frequently.
2. I found the system unnecessarily complex.
3. I thought the system was easy to use.
4. I think that I would need the support of a technical person to be able to use this system.
5. I found the various functions in this system were well integrated.
6. I thought there was too much inconsistency in this system.
7. I would imagine that most people would learn to use this system very quickly.
8. I found the system very cumbersome to use.
9. I felt very confident using the system.
10. I needed to learn a lot of things before I could get going with this system.

### Other questions

1. What course are you teaching?
2. What aspect of the web application did you like the most?
3. Which aspect of the web application did you like the least?
4. What would you improve about the web application?
5. Do you think the web application could be useful for your learners?
6. Which learners do you think the web application is best suited for? (Course, etc.)
7. Would you use this web application in your classes?
8. Do you use any application to practice the ABN method in your classes? (a) If yes, please indicate the name of the application you use.
9. Do you use any apps with your learners in other subjects?
  - If yes, please indicate which one.
10. Any comment you would like to mention.
